# Phosphorylation at Ser289 Enhances the Oligomerization of Tau Repeat R2

**DOI:** 10.1021/acs.jcim.2c01597

**Published:** 2023-02-14

**Authors:** Viet Hoang Man, Xibing He, Fengyang Han, Lianjin Cai, Luxuan Wang, Taoyu Niu, Jingchen Zhai, Beihong Ji, Jie Gao, Junmei Wang

**Affiliations:** † Department of Pharmaceutical Sciences and Computational Chemical Genomics Screening Center, School of Pharmacy, University of Pittsburgh, Pittsburgh, Pennsylvania 15261, United States; ‡ Department of Neuroscience, The Ohio State University Wexner Medical Center, Columbus, Ohio 43210, United States

## Abstract

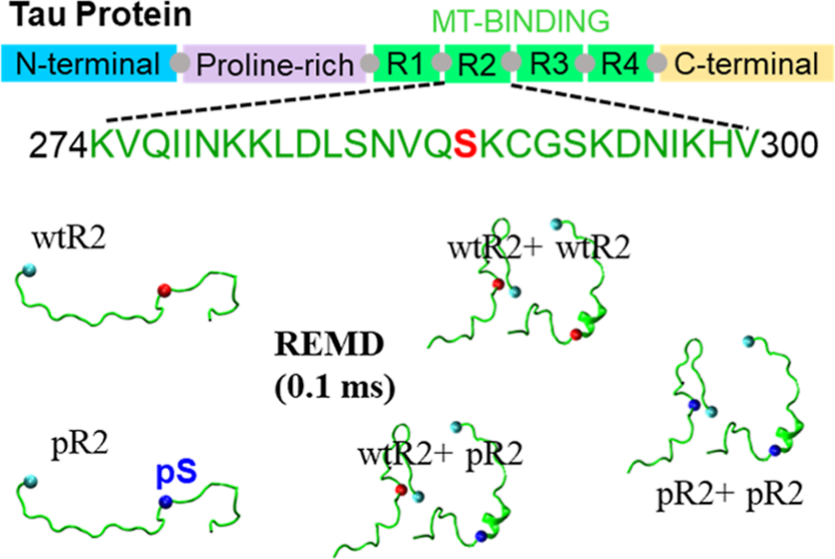

In tauopathies such as Alzheimer’s disease (AD), aberrant phosphorylation causes the dissociation of tau proteins from microtubules. The dissociated tau then aggregates into sequent forms from soluble oligomers to paired helical filaments and insoluble neurofibrillary tangles (NFTs). NFTs is a hallmark of AD, while oligomers are found to be the most toxic form of the tau aggregates. Therefore, understanding tau oligomerization with regard to abnormal phosphorylation is important for the therapeutic development of AD. In this study, we investigated the impact of phosphorylated Ser289, one of the 40 aberrant phosphorylation sites of full-length tau proteins, on monomeric and dimeric structures of tau repeat R2 peptides. We carried out intensive replica exchange molecular dynamics simulation with a total simulation time of up to 0.1 ms. Our result showed that the phosphorylation significantly affected the structures of both the monomer and the dimer. For the monomer, the phosphorylation enhanced ordered–disordered structural transition and intramolecular interaction, leading to more compactness of the phosphorylated R2 compared to the wild-type one. As to the dimer, the phosphorylation increased intermolecular interaction and β-sheet formation, which can accelerate the oligomerization of R2 peptides. This result suggests that the phosphorylation at Ser289 is likely to promote tau aggregation. We also observed a phosphorylated Ser289-Na^+^-phosphorylated Ser289 bridge in the phosphorylated R2 dimer, suggesting an important role of cation ions in tau aggregation. Our findings suggest that phosphorylation at Ser289 should be taken into account in the inhibitor screening of tau oligomerization.

## Introduction

Alzheimer’s disease (AD), the most common type of dementia, is one of the most common tauopathies. AD patients suffer from clinical manifestations including memory loss, disorientation, loss of motivation, language decay, difficulties in managing self-care, and behavioral issues.^1^ The number of people with dementia in the world is predicted to increase from 57.4 million in 2019 to 152.8 million in 2050.^2^ Although it has been 116 years since AD was first officially described,^3^ there is still no cure or effective treatment for AD yet, especially for the patients in the late stages. This may result from the complex etiology and lack of understanding of the pathogenesis of AD.

Tau protein, a “tubulin-associated unit”, plays an important physiological role in stabilizing and bundling axonal microtubules (MTs). It undergoes various post-translational modification forms, including phosphorylation, acetylation, glycosylation, methylation, nitration, SUMOylation, truncation, and ubiquitination.^4,5^ Among these modifications, phosphorylation is the most popular and of great interest. Tau regulates MTs through a phosphorylated/dephosphorylated reversible process in a healthy brain. However, abnormal phosphorylation can make tau lose affinity for MTs and presumably promote aggregation. Tau proteins aggregate into paired helical filaments (PHF), which then assemble into insoluble neurofibrillary tangles (NFTs). NFTs, which are twisted fibers and a hallmark of neurodegenerative tauopathies such as AD, disrupt normal cellular functioning and result in the death of the nerve cells.^6^ Intriguingly, recent solid evidence has showed that tau oligomers (TOs), formed in the early stage of tau aggregation, are more toxic than NFTs. TOs cause neuronal damage, leading to neurodegeneration and traumatic brain injury.^7−9^ Therefore, it is of great interest to investigate tau oligomerization and the impact of abnormal phosphorylation on the process.

Tau exists as six isoforms with the length varying from 352 to 441 residues. A full-length adult tau (441 residues of length) includes an N-terminal projection, a proline-rich domain, an MT-binding region, and a C-terminal domain.^10^ The N-terminal and C-terminal domains are significantly disordered.^11^ The MT-binding region is more structured and contains three or four imperfect sequence repeats, namely, R1, R2, R3, and R4. These repeats play important roles not only in the binding to MTs but also in the pathological aggregation of Tau proteins.^12−14^ The full-length adult tau has 85 potential phosphorylation sites including 45 serine, 35 threonine, and 5 tyrosine residues. Of these sites, about 20 residues undergo phosphorylation in tau from both healthy and AD brains, while about another 40 occur in the tau of AD brain only.^15^ The different phosphorylation sites and their combinations have different effects on tau aggregation. Phosphorylation at Thr175 leads to the fibril formation of tau and facilitates cell death.^16^ Pseudo-phosphorylation at Thr17, Thr212, Ser202, and Thr205 promotes tau filament formation.^17,18^ Ser422 pseudo-phosphorylation increases tau oligomerization.^19^ Triple phosphorylation at Ser202/Thr205/Ser208 can lead to rapid tau aggregation,^20^ while some phosphorylation sites such as Ser214, Ser262, and Ser305 inhibit tau aggregation.^21,22^

Ser289 residue is one of the 40 phosphorylation sites that are not found in a healthy brain but in AD brain only.^15^ Notably, this phosphorylation site has been determined in insoluble PHF from AD brain,^23^ suggesting that it promotes tau aggregation. However, the role of this phosphorylation site in tau aggregation has not been investigated in any previous studies. In this work, we used replica exchange molecular dynamics (REMD) simulation to investigate the impact of phosphorylated Ser289 on tau aggregation, particularly the oligomerization of tau R2 peptides. MD simulation plays an important role in amyloid aggregation studies.^24^ It is widely applied in the investigation of amyloid oligomerization and the characterization of soluble amyloid oligomer structures at the atomic level, which are still grand challenges to the currently available experimental technologies.^25−31^ REMD, an enhanced sampling method, can accelerate the exploration of free energy landscapes, resulting in the atomistic pictures of the oligomerization to be captured accurately. Currently, it is too time- and computer resource consuming to consider the full-length tau proteins in an MD simulation study of tau oligomerization due to its big size and the disordered nature of the protein. Instead, tau fragments such as tau repeats, PHF6 (306VQIVYK311) and PHF6* (275VQIINK280) peptides, are used frequently since they are the essential segments for the fibrillization and somehow can represent the full-length tau.^11,32−39^ We performed REMD simulations for five systems, two monomeric ones and three dimeric ones, with a total simulation time of about 0.1 ms. We characterized structures of R2 monomers and dimers for wild-type, phosphorylated, and wild-type and phosphorylated mixed cases. Our result revealed the effect of the phosphorylation Ser289 on the conformation as well as the oligomerization process of R2 peptides. The phosphorylation enhanced intramolecular and intermolecular interactions of the peptides and promoted the β-sheet formation of R2 dimers. It revealed a critical role of phosphorylated Ser289 in the oligomerization of tau proteins and recommended further experimental investigations. Additionally, our result also demonstrated the important role of cations in tau oligomerization. Therefore, our work provides a kinetic basis for studying AD progression and a structural basis for rational design of druglike molecules that can inhibit the pathological tau oligomerization.

## Materials and Methods

### System Design

To prepare initial structures for the REMD simulations of monomeric and dimeric systems, we constructed monomeric structural databanks using the following steps. Tau R2 peptide was taken from an experimental structure of R2–MT complex (PDB code 6CVN).^40^ The peptide was capped by acetyl (ACE) at the N-terminus and *N*-methylamine at the C-terminus, namely, wtR2. The peptide wtR2 was then placed into an octahedron box of explicit water, and the smallest distance from the peptide to the box border was larger than 1.2 nm. Next, this system, wtR2 in waters, underwent a 500 ns *NPT* simulation at a pressure of 1 atm and temperature of 300 K. From the last 400 ns trajectory, a clustering analysis with a cutoff of 0.35 nm was applied to the peptide structures. 80 clusters, which have the largest populations, were deposited on the monomeric structural databank. The phosphorylated Ser289 R2 peptide (pS289R2) was obtained by phosphorylating the experimental R2 peptide at Ser289 residue, and a similar procedure as that for the wtR2 case was carried out for the pS289R2 peptide to generate another monomeric structural databank.

For an REMD simulation of monomeric system, 42 monomers, which were used as initial structures, were randomly selected from the monomeric structural databank. Each monomer was placed into an octahedron box of explicit water with a box size of 7.5 nm. Each monomeric system contains 10,300 water molecules. An REMD simulation of the dimeric system was designed using the following steps: (i) two monomers were randomly taken from related monomeric structural databanks and placed close to each other, and the minimum distance between two monomers was smaller than 0.33 nm but greater than 0.13 nm. Note that the minimum distance is the smallest distance between two atoms coming from two different monomers; (ii) 49 dimers were built and used as the initial structures for the subsequent MD simulations; (iii) each of the dimers was placed into an octahedron box of ∼13,000 explicit water with a box size of 8.1 nm. Sodium cations (Na^+^) and chloride anions (Cl^–^) were added to all the simulation systems to have a neutral net charge and 0.15 M of salt concentration. The above box sizes ensure the minimum distance between any atom of the peptides, and the edges of the water box was at least 10 Å. In total, we built two REMD monomeric systems and three REMD dimeric systems. The monomeric systems are wild type R2 (wtR2) and phosphorylated Ser289 R2 (pS289R2). The dimeric systems include two wild-type R2 peptides (wtR2 + wtR2), one wild-type R2 peptide, one phosphorylated Ser289 R2 peptide (wtR2 + pS289R2), and two phosphorylated Ser289 R2 peptides (pS289R2 + pS289R2).

### Simulation Details

The GROMACS 2018 package^41^ was employed for all simulations. The peptides were described by Charmm36m force field,^42^ which is currently the most suitable to simulate amyloid aggregations based on a series of benchmarking simulation tests.^39,43^ The TIP3P model^44^ was used to represent explicit water molecules. The solvated systems were minimized using the steepest descent method and were equilibrated for 1 ns at a constant pressure of 1 atm. The pressure and temperature of the simulations were controlled using the Berendsen coupling method^45^ with a relaxation time of 3 ps and the Bussi–Donadio–Parrinello velocity scaling method^46^ with a relaxation time of 1 ps, respectively. The equations of motion were integrated using a leap-frog algorithm^47^ with a time step of 2 fs. The REMD temperatures ranged from 298 to 400 K, which were covered by 42 replicas of the monomeric systems and 49 replicas of the dimeric systems. The temperatures of the 42 replicas of a monomeric system were 298.00, 300.12, 302.26, 304.42, 306.60, 308.79, 311.00, 313.23, 315.48, 317.75, 320.03, 322.33, 324.65, 326.99, 329.34, 331.72, 334.11, 336.51, 338.94, 341.38, 343.84, 346.31, 348.80, 351.31, 353.84, 356.38, 358.94, 361.51, 364.10, 366.70, 369.32, 371.96, 374.61, 377.28, 379.96, 382.65, 385.36, 388.09, 390.82, 393.58, 396.34, and 399.12 K. The temperatures of the 49 replicas of a dimeric system included 298.00, 299.87, 301.75, 303.65, 305.56, 307.48, 309.42, 311.37, 313.34, 315.32, 317.32, 319.32, 321.35, 323.38, 325.43, 327.50, 329.57, 331.66, 333.77, 335.89, 338.02, 340.16, 342.32, 344.49, 346.67, 348.87, 351.08, 353.30, 355.54, 357.78, 360.04, 362.32, 364.60, 366.90, 369.21, 371.53, 373.86, 376.20, 378.56, 380.93, 383.31, 385.70, 388.10, 390.51, 392.93, 395.36, 397.81, 400.26, and 402.73 K. Exchanges between two replicas were attempted every 2 ps, leading to a mean acceptance ratio of 20%. 300 and 500 ns *NPT* sampling simulations were subsequently conducted for each replica of monomeric and dimeric systems, respectively. They result in 12.6 and 24.5 μs of simulations for each monomeric and dimeric system and 98.7 μs in total for all five systems. The LINCS algorithm^48^ was used to constrain the lengths of all covalent bonds. The van der Waals forces were calculated with a cutoff of 10 Å, and the particle mesh Ewald method^49^ was employed to treat the long-range electrostatic interactions. The nonbonded interaction pair list was updated every 5 fs using a cutoff of 10 Å. Periodic boundary conditions were applied to all the simulations. The REMD temperatures ranged from 300 to 400 K. Exchanges between two replicas were attempted every 2 ps, leading to a mean acceptance ratio of 20%.

### Data Analysis

The structures of R2 monomers and dimers sampled by MD simulations were characterized by intramolecular and intermolecular side chain–side chain contacts, intramolecular and intermolecular backbone hydrogen bond (H-bond), solvent accessible surface areas (SASA), gyration of radius (*R*_g_), and secondary structural contents. A side chain–side chain contact is formed if the minimum distance between two residue side chains is within 4.5 Å. An H-bond is formed if the acceptor–donor distance is within 3.5 Å and the acceptor–donor-H angle is less than 30°. The intramolecular interactions were calculated for two residues *i* and *j* when they were not nearest neighbors. The secondary structural contents were calculated by using the STRIDE algorithm (with helix content including 3–10 helix, π-helix, and α-helix),^50,51^ and H-bond, SASA, and *R*_g_ were calculated using GROMACS tools. The clustering analysis of the R2 dimers was performed using GROMACS tools with a cutoff of 8 Å.

## Results

**Figure 1:**
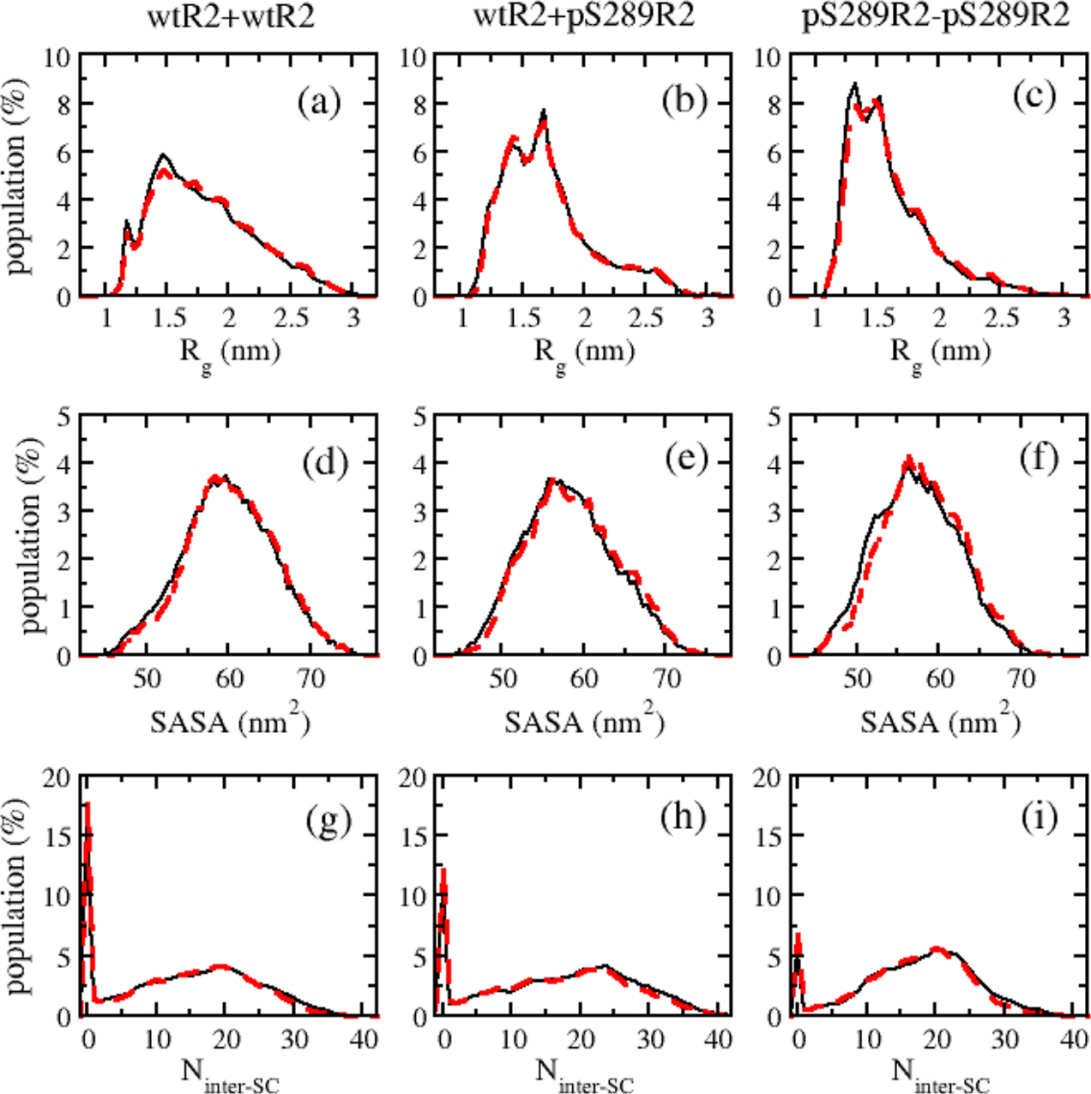
Distributions of gyration radius (*R*_g_) (a–c), SASA (d–f), and number of intermolecular side chain–side chain interactions (N_inter-SC_) (g–i) of dimeric R2 peptides. The results were obtained by two ensemble statistics at 310 K replica: with 300 ns spanning from 100 to 400 ns (red dashed lines) and with the last 400 ns (from 100 to 500 ns) (black solid lines).

Because of the disordered nature of amyloid peptides such as tau repeats, their monomeric and oligomeric structures are exceedingly diverse, resulting in a grand challenge to fully characterize them. Therefore, MD simulation of amyloid peptides requires not only conformation sampling to be converged but also the structural space to be covered. In this work, we employed REMD simulations to satisfy the first condition. Additionally, we used an innovative design of the simulations mentioned in the Methods section to cover a larger structural space than previous simulation studies which used only the same single structure for all replicas. By this way, the second condition is also satisfied. To ascertain the data analysis using only collected structures when the sampling reached convergence, we excluded the first 100 ns of all REMD simulations. Specifically, we used the last 400 ns of dimeric REMD simulations and the last 200 ns of monomeric REMD simulations for post-analysis. Figure 1 shows the distributions of *R*_g_, SASA, and intermolecular side chain–side chain interactions (N_inter-SC_) of dimers at 309.4 K replica in ensemble statistics, which were performed using both 300 ns spanning (from 100 to 400 ns) and the last 400 ns (from 100 to 500 ns). A similar analysis was performed for other structural parameters of dimers and monomers as shown in Figures S1 and S2 in the Supporting Information. Obviously, the two ensemble statistics resulted in very similar distributions for the reaction coordinates in all simulation systems. It suggests that the samplings were converged, and the simulation protocol was adequate. The temperature dependence of the reaction coordinates is shown in Figure S3 for the monomeric systems and Figure S4 for the dimeric systems. This result exhibited a similar trend compared to the systems between different replica temperatures. Therefore, in the rest of the text, unless mentioned explicitly, all observables are ensemble-averaged data at 311 K and within the simulation time of 100–300 ns for the monomeric systems and at 309.4 K and within the simulation time of 100–500 ns for the dimeric systems. These temperatures were selected because they are close to the physiological temperature.

### Impact of Phosphorylated Ser289 on the Conformation of Monomeric R2 Peptides

To examine how phosphorylation at Ser289 affects the structure of monomeric R2 peptides, we first characterized the monomers using overall structural parameters including SASA and *R*_g_. The distributions of SASA and *R*_g_ are shown in Figure S2, and the temperature dependences of those parameters are shown in Figure S3e,f, which demonstrated that the phosphorylated peptides were more compact than the wild-type one. For the *R*_g_ case, although the distributions of both peptides showed two maximum peaks at 1.0 and 1.2 nm (Figure S2a,b), the peaks of pS289R2 were higher than those of wtR2, and the *R*_g_ distribution of wtR2 had larger populations at the large values compared to the distribution of pS289R2. The averaged values of *R*_g_ were 1.36 and 1.25 nm for wtR2 and pS289R2, respectively. Similar to *R*_g_, the SASA distributions of both peptides had two peaks at 31 and 36.5 nm^2^ (Figure S2c,d), but the peak at 31 nm^2^ of pS289R2 was slightly higher than that of wtR2. The averaged values of SASA were 32.59 and 32.02 nm^2^ for wtR2 and pS289R2, respectively.

**Figure 2:**
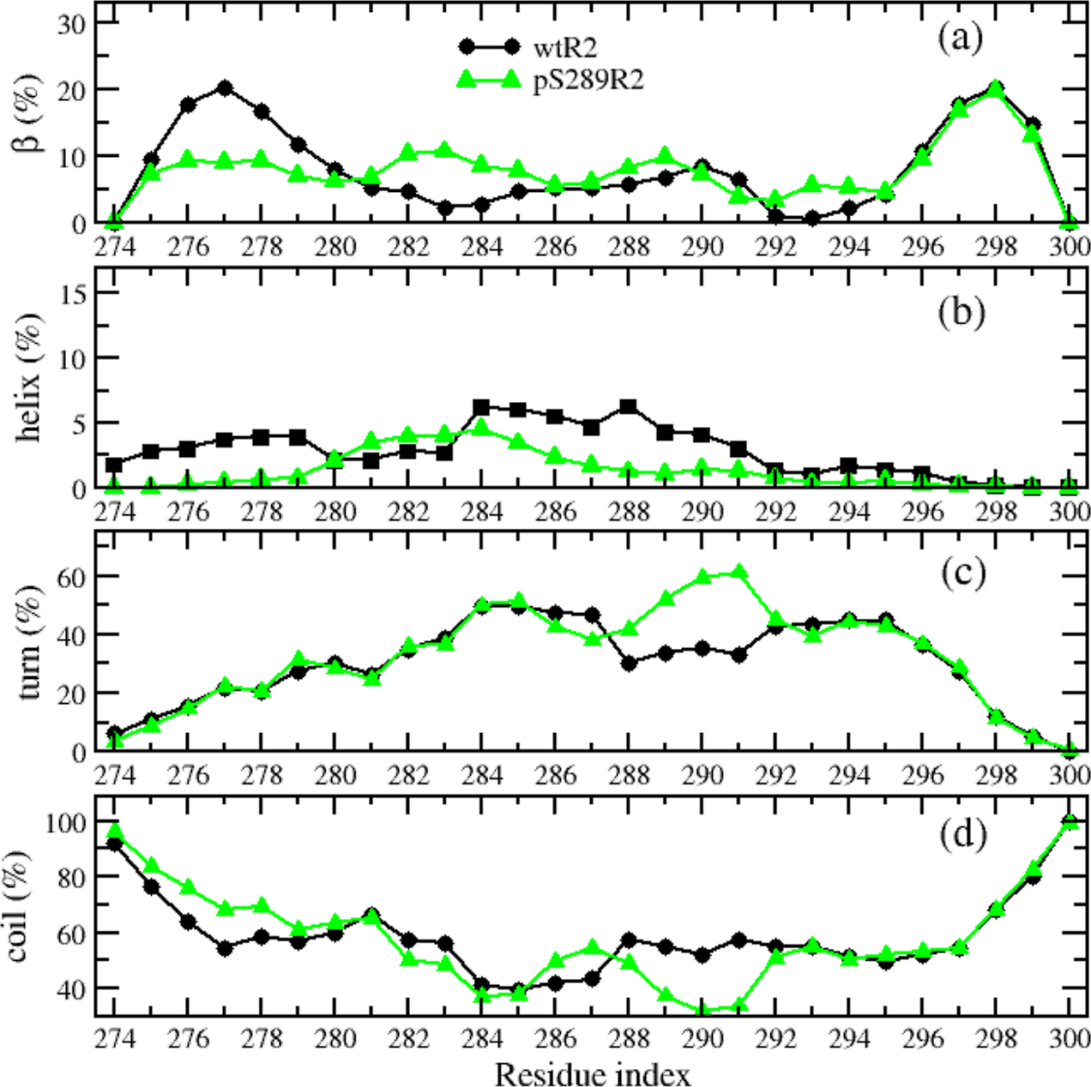
Secondary structure propensities of the amino acid of R2 peptides. The data include β (a), helix (b), turn (c), and coil (d) structures.

Next, we performed a secondary structural analysis of the monomers. As demonstrated in Figure S3a, the phosphorylation had little impact on the distribution of β-sheet content, and the average values were 8 and 7.8% for wtR2 and pS289R2, respectively. The average values of helix, turn, and coil contents were more distinct, which were 2.8, 30.2, and 59%, respectively, for wtR2, while those corresponding values were 1.3, 32.5, and 58.4% for pS289R2. The helix and coil contents of wtR2 were slightly greater than those of pS289R2, while the turn content of wtR2 was smaller than that of pS289R2. We also analyzed the secondary structural profile along the sequence, which provided more details of the phosphorylation impact. As shown in Figure 2, the distributions of secondary structures at the C-terminal (residues from 295 to 300) were almost identical for both types, while the β, helix, and coil profiles of the two peptides were significantly different at the N-terminal and middle residues. The turn profiles were only different at the residues spanning from 287 to 291, which are neighbors of the phosphorylated residue pS289. In summary, the phosphorylation reduced the helix propensity of most residues and the β propensity of N-terminal residues (275–280), while it enhanced the turn propensity of middle residues surrounding the pS289 residue.

**Figure 3:**
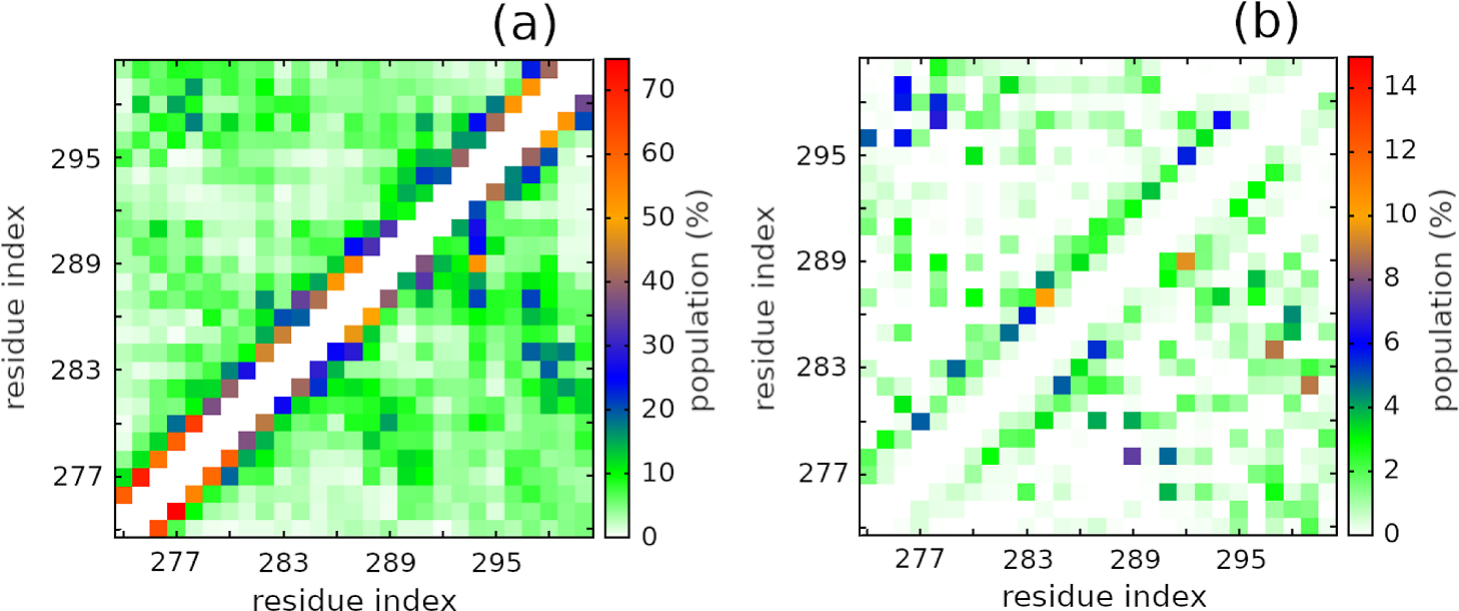
Intramolecular side chain–side chain (a) and hydrogen bond (b) interaction maps. In each panel, the upper and lower triangles represent the wild-type and pS289 R2 peptides, respectively.

We further investigated the intramolecular residue–residue interactions of the monomeric R2 peptides, as shown in Figure 3. Our result indicated that the intramolecular side chain–side chain and hydrogen bonds maps were significantly different for the wild-type and phosphorylated peptides. Overall, the phosphorylation increased intramolecular residue–residue interactions of R2 peptides. For wtR2, large interaction frequencies were revealed between the N-terminal and C-terminal residues, leading to a high β propensity of those residues (Figure 2a). On the contrary, the N-terminal residues demonstrated lower interaction frequencies for pS289R2, leading to an increase of disordered structural content (coil) and decrease of ordered structural contents (β and helix) as shown in Figure 2. On the other hand, C-terminal residues strongly interacted with middle residues, leading to a decrease of coil propensity and an increase of the turn and β propensities for the middle residues of pS289R2 (Figure 2a,c).

### Impact of Phosphorylated Ser289 on R2 Dimers

**Figure 4:**
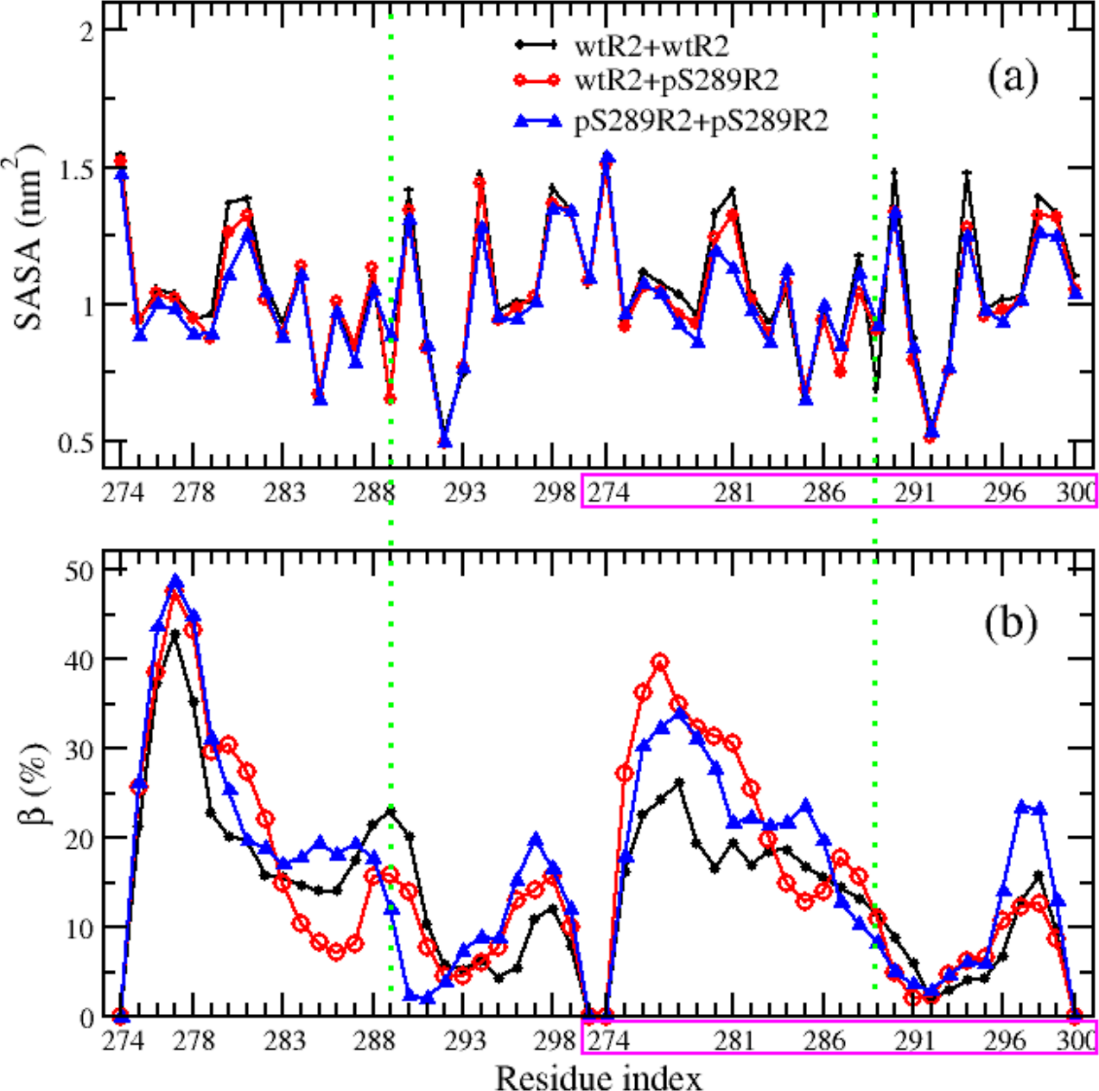
Distribution of SASA (a) and β content (b) along residues of R2 peptides in the dimeric systems. The second peptide of a dimer is marked by a pink rectangle.

In this section, we will examine the impact of the phosphorylation of pS289 on tau R2 dimers by characterizing the structures of three dimeric systems: wtR2 + wtR2, wtR2 + pS289R2, and pS289R2 + pS289R2. First, we considered the overall structural parameters including *R*_g_ and SASA. Our result indicated that the phosphorylation increased the compactness of R2 dimers (Figures 1a–f and S4e,f). The order of compactness followed pS289R2 + pS289R2 > wtR2 + pS289R2 > wtR2 + wtR2, and the averaged *R*_g_ values were 1.79, 1.70, and 1.59 nm for wtR2 + wtR2, wtR2 + pS289R2, and pS289R2 + pS289R2, respectively. The averaged SASA values followed the same trend, which were 59.94 nm^2^ for wtR2 + wtR2, 58.29 nm^2^ for wtR2 + pS289R2, and 57.39 nm^2^ for pS289R2 + pS289R2. The per-residue analysis of SASA shown in Figure 4a suggested that for most residues, the residue-based SASA is larger in wtR2 + wtR2 than in wtR2 + pS289R2 and pS289R2 + pS289R2 dimers; except for Ser289, the residues undergo phosphorylation. Interestingly, residues 280, 281, 290, 294, and 295, which have significantly larger SASA in the wtR2 + wtR2 dimer than in the phosphorylated dimers, are lysine, an amino acid with one positive charge. Moreover, pS289 was more exposed than S289 as we expected.

Subsequently, we analyzed the secondary structures of the R2 dimers. For wtR2 + wtR2, the secondary contents were 14.17, 1.87, 26.17, and 57.78 for sheet, helix, turn, and coil, respectively. For wtR2 + pS289R2, those secondary structure contents were 16.21, 1.52, 26.48, and 55.79% for the four aforementioned secondary structures correspondingly. As for pS289R2 + pS289R2, those values were 16.89% for sheet, 0.95% of helix, 28.16% for turn, and 54.00% for coil. Thus, the effect of phosphorylated S289 on the β content of R2 peptides in monomeric and dimeric systems was different. For the dimeric systems, phosphorylation increased the β-sheet content but decreased the coil content.

**Figure 5:**
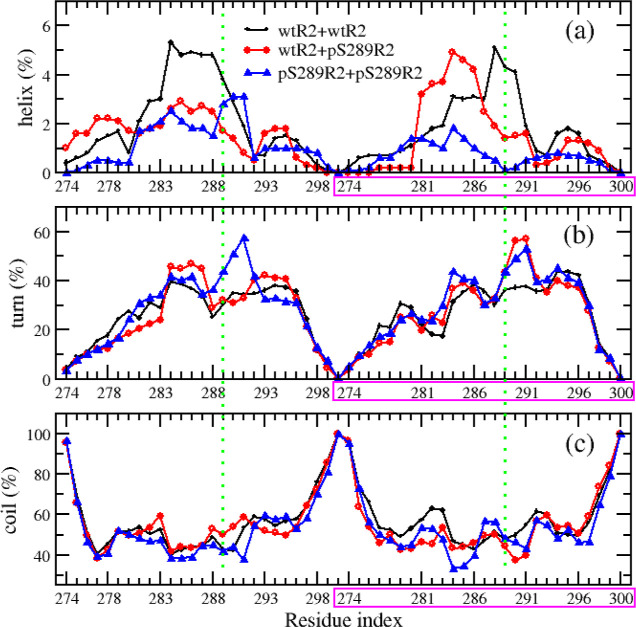
Distribution of SASA and helix (a), turn (b), and coil (c) contents along residues of R2 peptides in dimer systems. The second peptide of a dimer is marked by a pink rectangle.

The distribution of the secondary structural contents along residues is shown in Figures 4b and 5. This data showed that the β-sheet contents of N-terminal and C-terminal residues of the phosphorylated R2 were higher than corresponding residues of wild-type R2, while the β-sheet content of the middle residues of R2 peptides demonstrated different patterns. It is pointed out that the hexapeptide PHF6* (residues 275–280), an essential segment for tau fibrillization,^32,33^ was located at the N-terminal of the R2 peptide. Therefore, the phosphorylation may enhance fibrillization. The phosphorylation significantly changed the turn and coil propensities of the phosphorylated residue and its neighboring residues. Similar to the monomeric system, the phosphorylation increased turn propensity and slightly reduced coil propensity of those residues. This observation indicated that phosphorylation at S289 facilitated the formation of turn structures. The average turn propensity of residues 289, 290, and 291 was 31.79 and 43.08% for the wild-type and phosphorylated dimeric peptides, respectively. The coil propensity of those residues was 48.85 and 45.8% for the wild-type and phosphorylated dimeric peptides, respectively.

**Figure 6:**
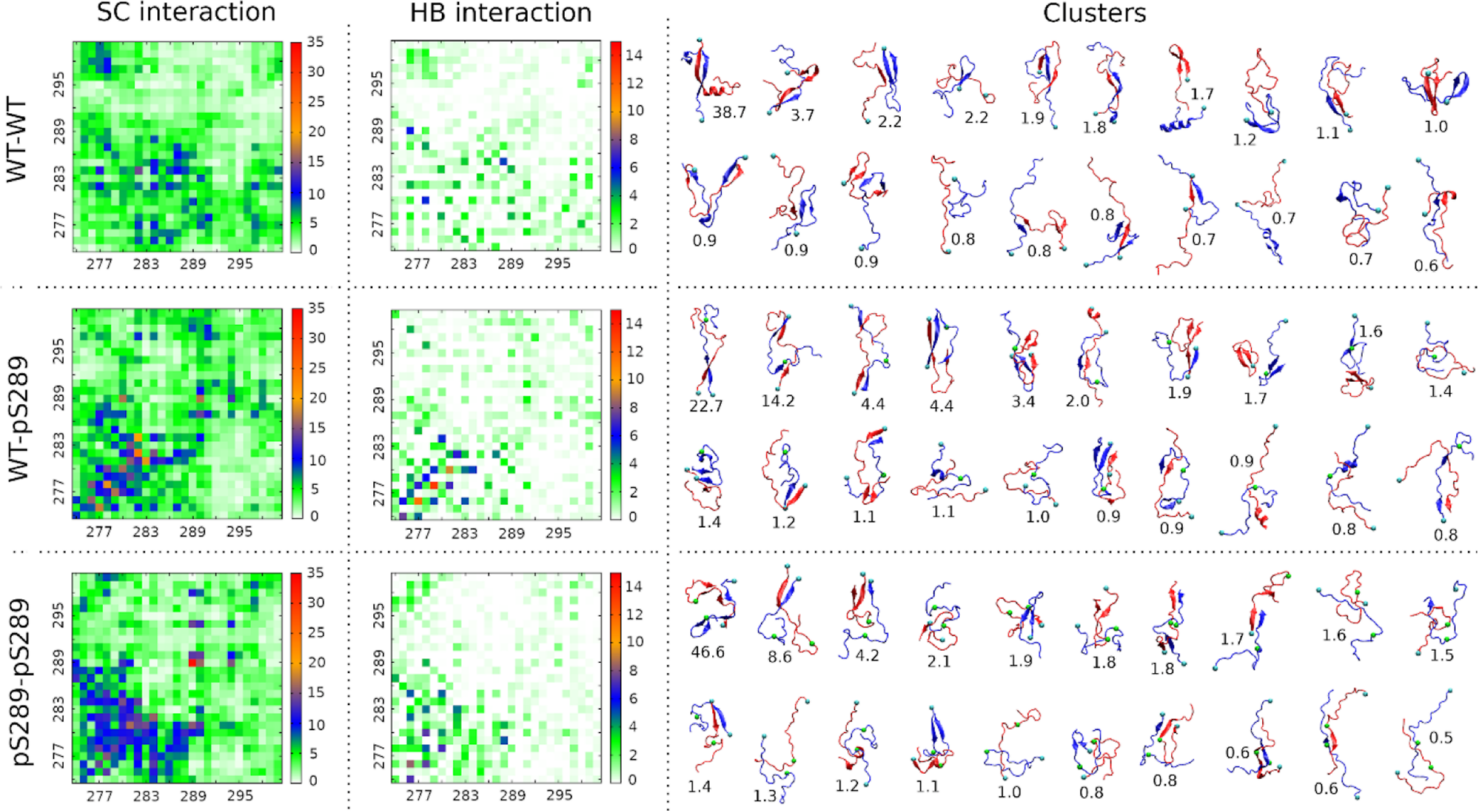
Intramolecular side chain–side chain interaction maps, hydrogen bond interaction maps, and structural clusters of R2 dimers. The population size of a cluster is indicated by the number below the representative structure.

**Figure 7:**
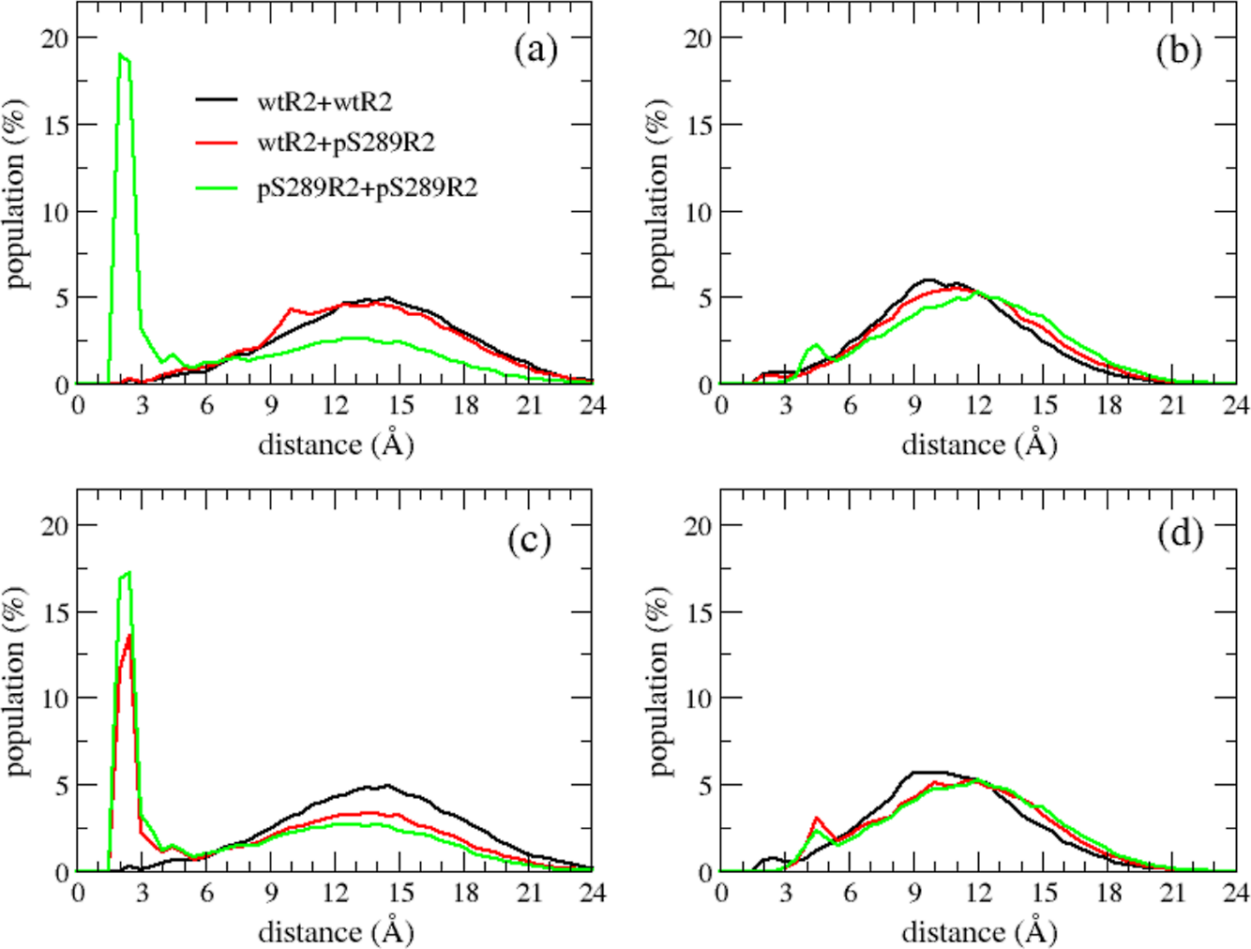
Populations of ion-Ser/pSer distance in WT–WT (black lines), WT–pS289 (red lines), and pS289–pS289 (green lines) dimeric systems. (a) Distances between sodium ions and the first R2 peptide, (b) distances between chloride ions and the first R2 peptide, (c) distances between sodium ions and the second R2 peptide, and (d) distances between chloride ions and the second R2 peptide.

In an oligomerization process, proteins/peptides interact with each other and form oligomers. Therefore, intermolecular interaction analysis is important to characterize the oligomeric structure as well as to reveal the oligomerization mechanism. Figure 6 shows the intermolecular side chain–side chain and hydrogen bond interaction architecture/layout of R2 dimers. It clearly demonstrated that the phosphorylation dramatically changed the intermolecular interaction. Intermolecular residue–residue interactions of wtR2 + pS289R2 and pS289R2 + pS289R2 dimers occurred more frequently than those of wtR2 + wtR2. In the wtR2 + wtR2 system, interactions frequently occurred between middle residues of the first peptide and middle residues of the second peptide, N-terminal residues of a peptide and C-terminal residues of the other peptide, and N-terminal residues of a peptide and middle residues of the other peptide. Particularly, the strong interaction residue pairs were 276–286, 277–282, 277–284, 278–292, 282–284, 284–284, and 284–284. In the wtR2 + pS289R2 system, interactions frequently happened between N-terminal residues of the wild-type peptide and N-terminal residues of the phosphorylated peptide, which contains the PHF6* hexapeptides. The interaction pattern facilitated the two PHF6* fragments forming a parallel β-sheet structure in wtR2 + pS289R2. Intriguingly, the pS289 residue of the phosphorylated R2 peptide strongly interacted with K280 and K290 residues of the wild-type peptide. These strong interactions are expected as lysine and phosphorylated serine bear opposite net charges. For the pS289R2 + pS289R2 system, likewise, strong intermolecular interactions occurred between the residues of the two PHF6* fragments. Additionally, the residues of the PHF6* fragments also frequently interacted with the middle peptide fragment spanning from 282 to 290 residues. Surprisingly, the strongest interaction was formed by two phosphorylated residues, which are both negatively charged. To explain this interesting observation, we further investigated the interactions between phosphorylated residues and sodium and chloride ions. Figure 7 shows the population of minimum ion–S289/pS289 distance in the dimeric systems. As observed, the populations of Cl-S289 and Cl-pS289 distances were similar, while populations of Na^+^-S289 and Na^+^-pS289 distances were vastly different. The population of Na^+^-pS289 distance has a high peak at small values of the distance (2–3 Å), while the Na^+^-S289 distance is more popular at the large values (9–18 Å). This result implied that there existed a strong interaction between the Na^+^ ion and the phosphorylated residue, resulting in the formation of the pS289-Na-pS289 bridge (Figure S5). The pS289-Na^+^-pS289 bridge occurred in the 16% of population sampled in MD simulations for the pS289R2 + pS289R2 dimeric system.

To identify representative structures of R2 dimers, which may serve as receptor targets for screening inhibitors of tau oligomerization, we performed clustering analysis on the sampled dimeric R2 structures using a cutoff of 8 Å. The numbers of clusters were 1119 for wtR2 + wtR2, 787 for wtR2 + pS289R2, and 417 for pS289R2 + pS289R2 systems. Our result suggested that those structures were diverse, reflecting the fact that tau is an intrinsically disordered protein. Additionally, with the same cutoff applied in clustering analyses, the number of clusters was inversely proportional to the number of phosphorylated R2 peptides, indicating that phosphorylation promotes the oligomerization of R2 peptides. The representative structures for the first 20 clusters are shown in Figure 6.

## Discussion

In tau pathologies such as AD, tau is aberrantly phosphorylated and lose affinity to MTs. This results in tau mislocalization from the axon to the cytoplasm and dendrites, which is considered as a primary lesion, with subsequent loss of function.^9,52−54^ The mislocalized tau proteins aggregate into consequent pathogenic forms including soluble oligomers, insoluble fibrils, and NFTs.^6,9,54−56^ All these tau aggregates are toxic to the brain, but the oligomers are a major contributor in tau pathologies.^7−9,54,56^ Therefore, investigation on tau oligomerization and characterization of TOs, regarding abnormal phosphorylation, is of great interest to support the therapeutic development of tau aggregation-related diseases. Recent studies have paid more attention to low-weight TOs such as dimers and trimers, which are considered the most hazardous/lethal form of TOs.^56^ Although TOs can be detected experimentally, their metastable structures are difficult to be resolved/characterized using the current experimental technologies. On the other hand, MD simulation, which is able to study the oligomerization process at the atomic level, complements experiments to solve the great challenge of studying tau oligomerization. However, we also want to point out that it is still challenging to study oligomerization using the full-length even truncated tau protein with atomistic MD simulation. Fortunately, some tau fragments, especially tau repeats that play a vital role in the aggregation, can be applied to represent the full-length tau in oligomerization studies to some degree. Through those MD simulations, one is expected to obtain the atomistic pictures of the oligomeric structures and the kinetic details of the oligomerization process.

In earlier days, a number of studies were carried out to investigate monomeric and dimeric structures of tau repeats (R1–R4) using MD simulation.^36−38,57^ These studies considered monomers and dimers of the four wild-type tau repeats as well as some undergoing mutation on the R3 repeat. However, to the best of our knowledge, there is no report on studying the phosphorylated R2 repeat. The structures of the repeats reported in the literature were different. For example, for the R3 monomer, the secondary structure contents of β-sheet, helix, turn, and coil were correspondingly 1.58, 18.3, 28.91, and 51.11% according to Liu et al.’s study;^36^ 18.3, 0.5, 6.2, and 75% reported by He et al.;^38^ and 21, 5, 34, and 40% reported by Dong et al.^37^ The different results may come from different simulation protocols applied in those studies. Liu et al. applied REMD simulation using the Amber ff99SB force field^58^ for protein and an implicit water model, He et al. employed all-atom discrete MD simulation with a Charmm force field^59^ for protein and an implicit water model, and Dong et al. performed REMD simulation with the Amber ff99SB-ILDN force field^60^ for protein and in explicit TIP3P water.^44^ Despite the fact that those works provided the convergence of simulation data, the force fields and implicit water models are not the best choices for a tau simulation study.^39^

In this work, we investigated monomeric and dimeric structures of tau R2 repeat, which were included in He et al.’s study, using intensive REMD simulation with the Charmm36m force field^42^ for protein and explicit TIP3P water. Note that Charmm36m is the most suitable force field for tau simulation study according to benchmark studies.^39,61^ In comparison with He et al.’s study, the structures of monomeric and dimeric R2 repeats in this study have higher β and turn contents and lower helix and coil contents. For the R2 monomer, we reported that the secondary structure contents of β-sheet, helix, turn, and coil were correspondingly 8, 2.8, 30.2, and 59%, which are quite different from those reported by He et al., which were 4.5, 9.2, 8.4, and 77.9%. For the R2 dimer (wtR2–wtR2), we reported secondary structure contents of β-sheet, helix, turn, and coil of 14.17, 1.87, 26.7, and 57.78%, which are are also different from those reported by He et al. (10.6, 7.2, 7.5, and 74.9%). The different results between our and Huan He et al.’s works may be caused by the different force fields and water models used in the simulations: we applied Charmm36m force field and explicit TIP3P water, while He et al. utilized an old Charmm force field and an implicit solvent. Previous CD experiment studies showed that the structure of R2 repeat contained about 30% of β-sheet, 10% of helix, and 70% of turn and coil contents. Therefore, our result is more consistent with the experimental finding as the β-sheet content is much higher than the helix content in our study. Despite the above significant difference, both works obtained the following common results. First of all, the β-sheet content of the R2 dimer is about 2 times higher than that in the monomer, and the β-sheet content increase mainly occurred at the C-terminal and N-terminal of the R2 peptides. Second, the helix and coil contents were reduced from monomeric to dimeric R2 peptide.

We performed large-scale REMD simulations using the computing resources with Intel(R) Xeon(R) CPU E5-2620 v3@2.40GHz provided by the Center for Research Computing (CRC) at the University of Pittsburgh. Each replica was assigned 8 CPU cores, resulting in 336 and 392 cores to be used for each monomeric and dimeric systems, respectively. The simulation speeds were 24 ns/day for monomeric systems and 18 ns/day for dimeric systems. Therefore, 100,800 CPU hours (SUs) were consumed running 300 ns MD simulations for a monomeric system, and 261,333 SUs were consumed running 500 ns MD simulations for a dimeric system. The total consumption of the computing resource was about 985000 Sus for this study.

Tau repeat R2 contains two abnormal phosphorylation sites: 289 and 293; it is interesting to investigate the phosphorylation effect on tau oligomerization for three different scenarios, that is, phosphorylation on S289, phosphorylation on S293, and phosphorylation on both S289 and S293. In this work, we only focused on phosphorylation on S289 due to the long duration of the study and the massive computer resource needed for REMD as discussed above. The other two scenarios, single phosphorylation on S289 and double phosphorylation on both sites, will be investigated in the future.

## Conclusions

Abnormal phosphorylation is a primary cause of tau pathologies. It not only affects Tau–MT binding but also impacts tau aggregation. Numerous possible phosphorylation sites of the full-length tau proteins require a huge work to understand their roles in tau-related diseases. The phosphorylation on various sites may trigger different effects on tau aggregation; some can accelerate,^20^ and others can inhibit the process.^21,22^ In this work, the impact of the abnormal phosphorylation at Ser289 on the monomeric and dimeric structures of R2 repeat is investigated for the first time, to the best of our knowledge. We applied enhanced sampling simulation (REMD), to obtain structures of the wild-type and phosphorylated R2 monomers and dimers described by a suitable force field (Charmm36m) and in explicit water. Our result on structures of the wild-type monomer and dimer agreed with previous experiment findings as it predicted β content to be higher than helix content. More importantly, we revealed the multiple roles of phosphorylated Ser289 on the R2 monomer and dimer. For the monomer, the phosphorylation increased intramolecular interaction and structural compactness and supported an ordered–disordered structural transition. For the dimer, the phosphorylation increased intermolecular interactions and enhanced β-sheet formation. Overall, our results suggest that the phosphorylation accelerates the oligomerization of R2 peptides, and consequently, it may promote tau aggregation. Furthermore, we also found the formation of a pS289-Na^+^-pS289 bridge, thus uncovering the essential role of cations in the oligomerization of the phosphorylated R2 repeat. Our findings suggest that phosphorylation at Ser289 should be taken into consideration when screening the inhibitors of tau oligomerization.

## Data and Software Availability

All the data were collected by running MD simulations with the GROMACS 2018 software package.

## Supporting Information Available

Distributions of the β-sheet content, coil content, and intermolecular hydrogen bonds (N_inter-HB_) of dimeric R2 peptides; distributions of *R*_g_ SASA of monomeric R2 peptides; secondary structures, SASA, and *R*_g_ of R2 monomers at different temperatures; secondary structural contents, SASA, and *R*_g_ of R2 dimers at different temperatures; and representative pS289-Na^+^-pS289 bridge formed in the pS289R2 + pS289R2 dimer (PDF)
