# Supplementary material for: Phosphorylation at Ser289 Enhances the Oligomerization of Tau Repeat R2
Source: J Chem Inf Model. 2023 Feb 14;63(4):1351–61. doi: 10.1021/acs.jcim.2c01597 (PMC10032562; doi:10.1021/acs.jcim.2c01597)
Supplement: Supplementary file 1 — ci2c01597_si_001.pdf [file ci2c01597_si_001.pdf]

SUPPORTING INFORMATION

Phosphorylation at Ser289 Enhances the Oligomerization of Tau Repeat R2

Viet Hoang Man,[1]* Xibing He,[1] Fengyang Han,[1] Lianjin Cai,[1] Luxuan Wang,[1] Taoyu Niu,[1] Jingchen Zhai,[1] Beihong Ji[1], Jie Gao,[2] and Junmei Wang[1]*

[1] *Department of Pharmaceutical Sciences and Computational Chemical Genomics Screening Center, School of Pharmacy, University of Pittsburgh, Pittsburgh, PA 15261, USA.*

[2] *Department of Neuroscience, The Ohio State University Wexner Medical Center, Columbus, OH 43210, USA.*

*E-Mails:

Viet Hoang Man: vhm3@pitt.edu
Junmei Wang: junmei.wang@pitt.edu

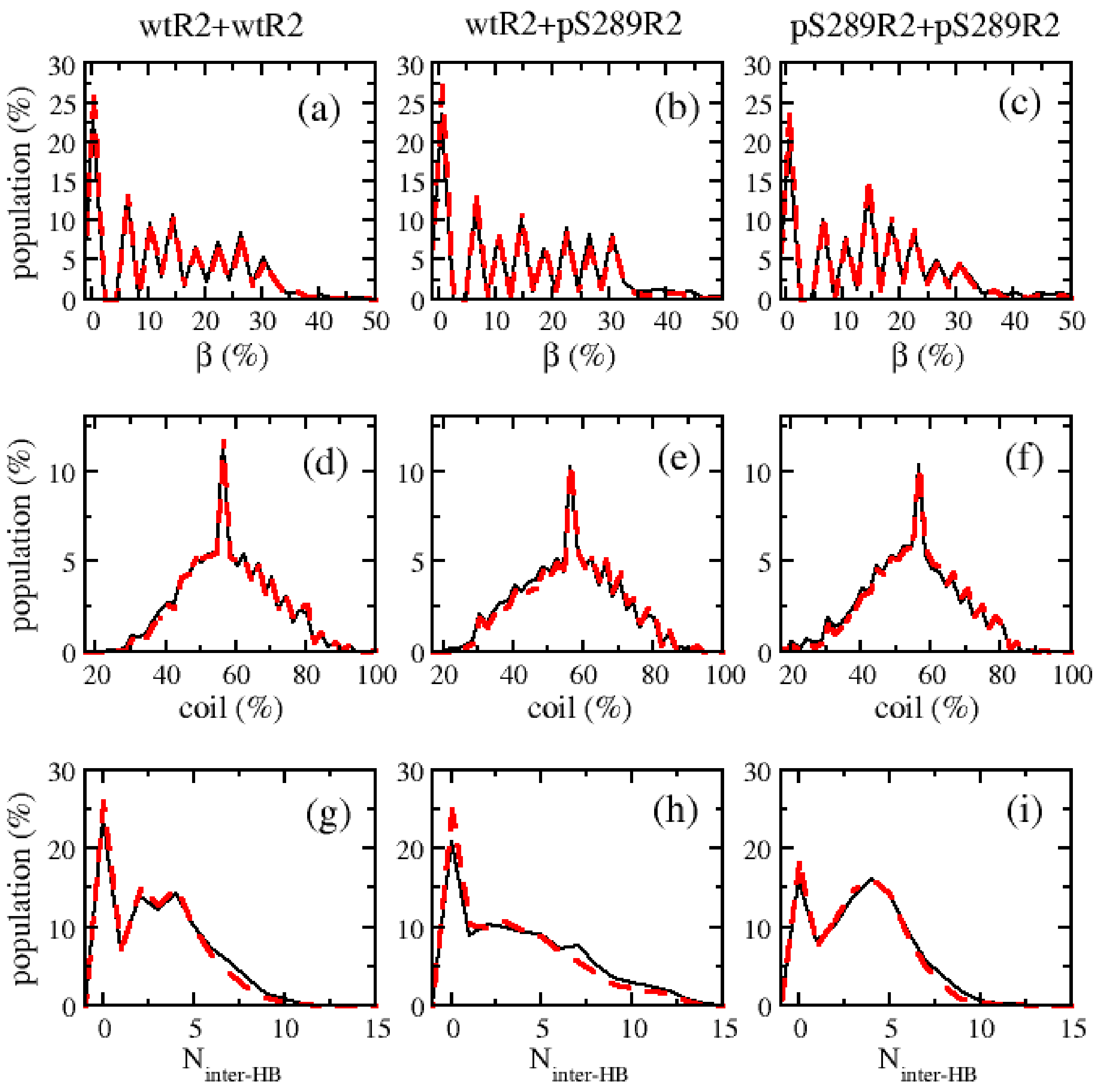

Figure S1: The distributions of β-sheet content (a-c), coil content (d-f), and intermolecular hydrogen bonds ($N_{inter\text{-}HB}$) (g-i) of dimeric R2 peptides. The results were obtained from two ensemble statistics at 309.4 K replica: with 300 ns spanning from 100 ns to 400 ns (red dashed lines); with the last 400 ns (from 100 ns to 500 ns) (black solid lines).

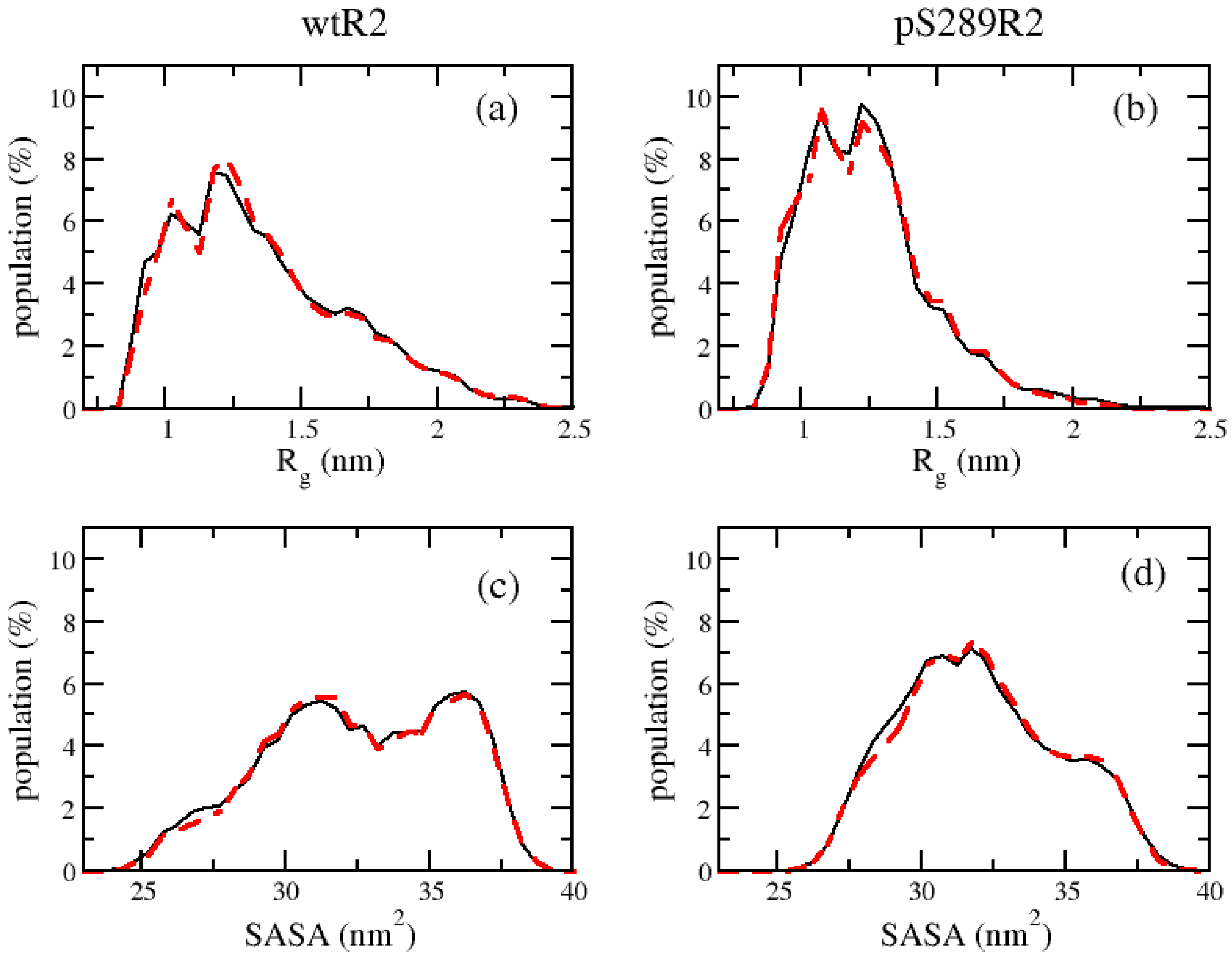

Figure S2: The distributions of gyration radius (R_g) (a, b) and solvent accessible surface area (SASA) (c, d) of monomeric R2 peptides. The results were obtained from two ensemble statistics at 311 K replica: with 150 ns spanning from 100 ns to 250 ns (red dashed lines); with the last 200 ns (from 100 ns to 300 ns) (black solid lines).

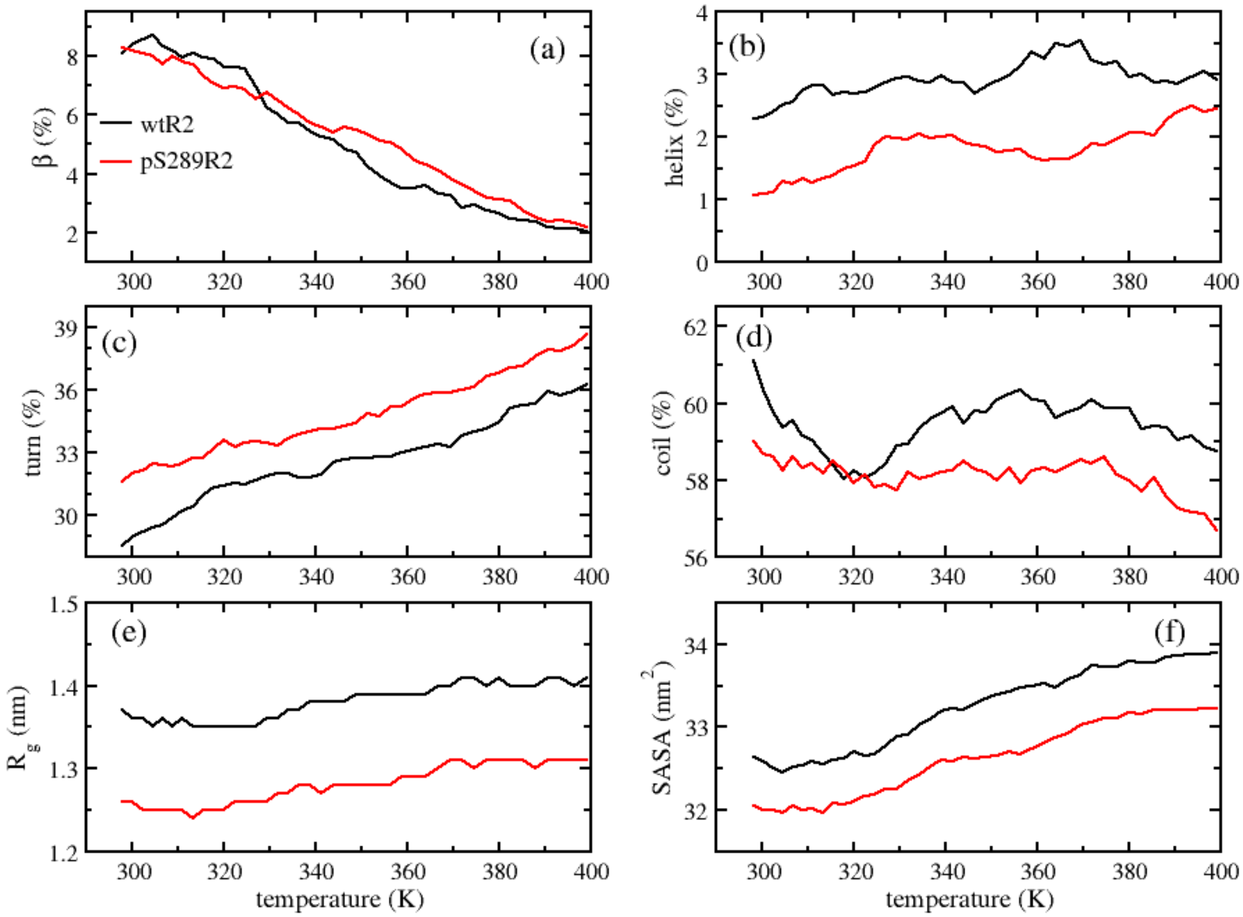

Figure S3: The β content (a), helix content (b), turn content (c), coil content (d), radius of gyrate (R_g) (e), and solvent accessible surface area (SASA) (f) of wild type (black lines) and pS289 (red lines) R2 monomers at different temperatures. The data is from the last 200 ns of each related replica.

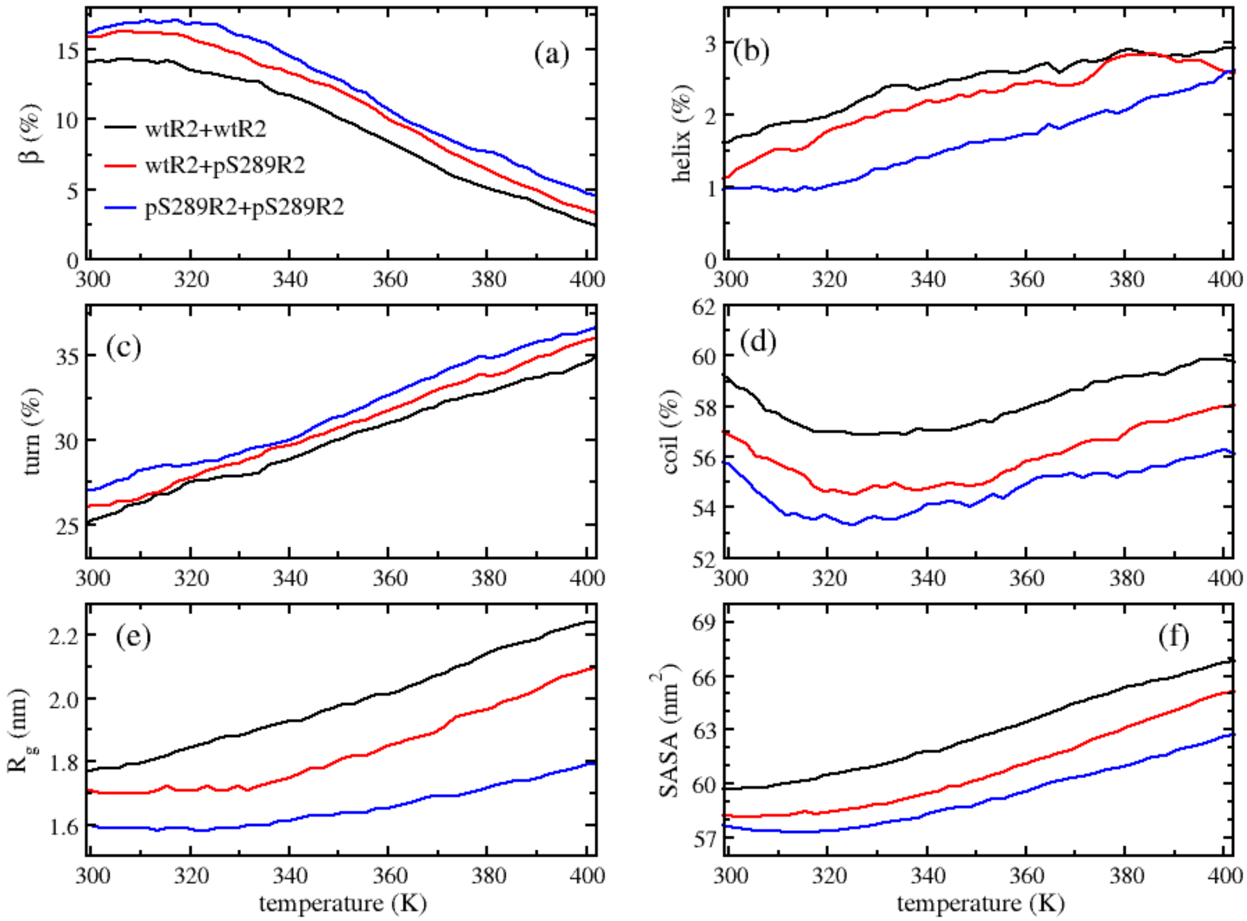

Figure S4: The β content (a), helix content (b), turn content (c), coil content (d), radius of gyrate (R_g) (e), and solvent accessible surface area (SASA) (f) of R2 dimers at different temperatures of REMD simulations. The data is from the last 400 ns of each related replica.

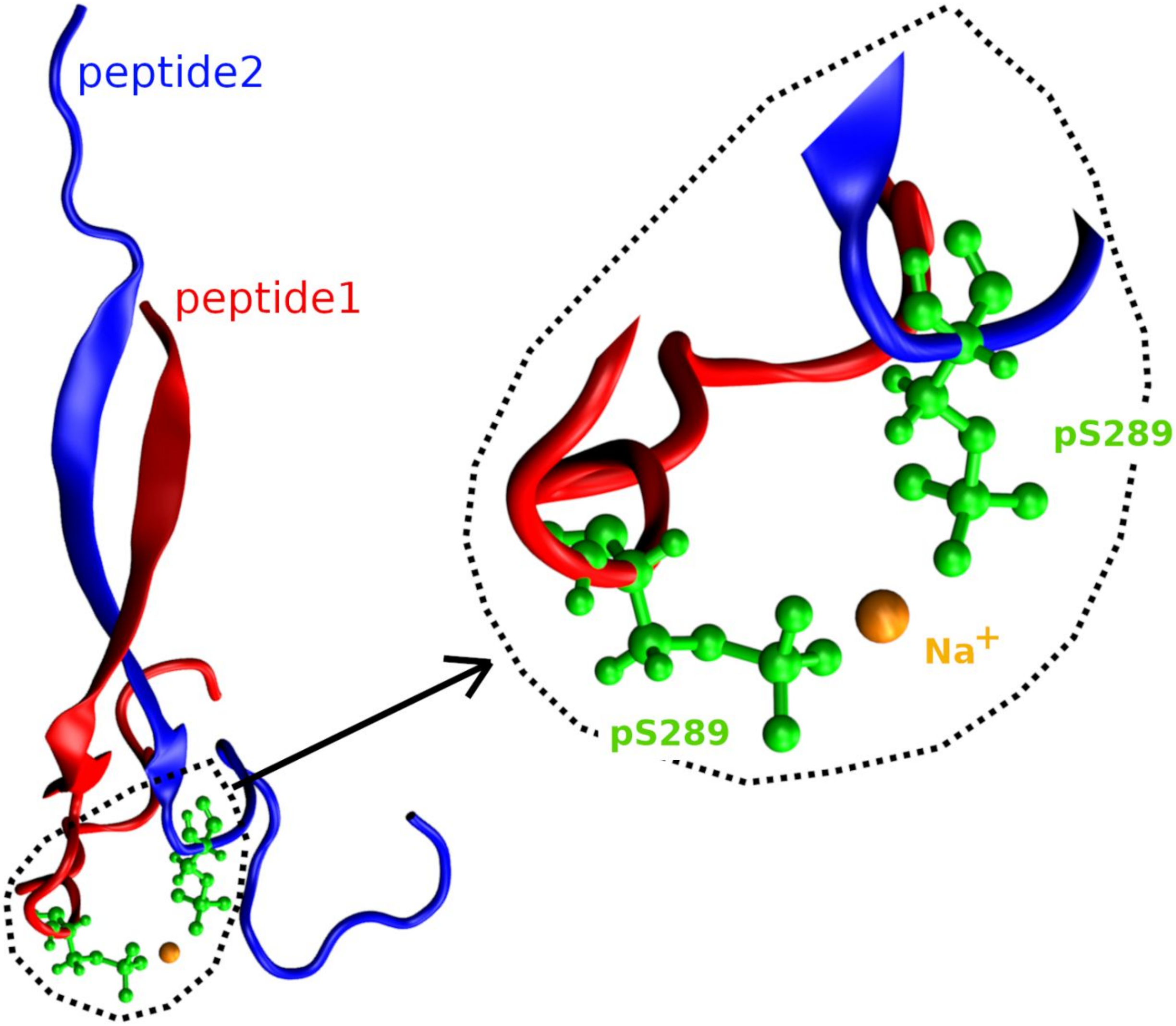

Figure S5: A representative pS289-Na^+-pS289 bridge formed in the pS289R2+pS289R2 dimer.
